# Supplementary material for: The causal role of circulating inflammatory markers in osteoporosis: a bidirectional Mendelian randomized study
Source: Front Immunol. 2024 Jul 18;15:1412298. doi: 10.3389/fimmu.2024.1412298 (PMC11291241; doi:10.3389/fimmu.2024.1412298)
Supplement: Supplementary file 1 [file DataSheet_1.zip › supplementary material/CIM-OSTEOPOROSIS /GCST90274791.csv_leave_one_out.pdf]

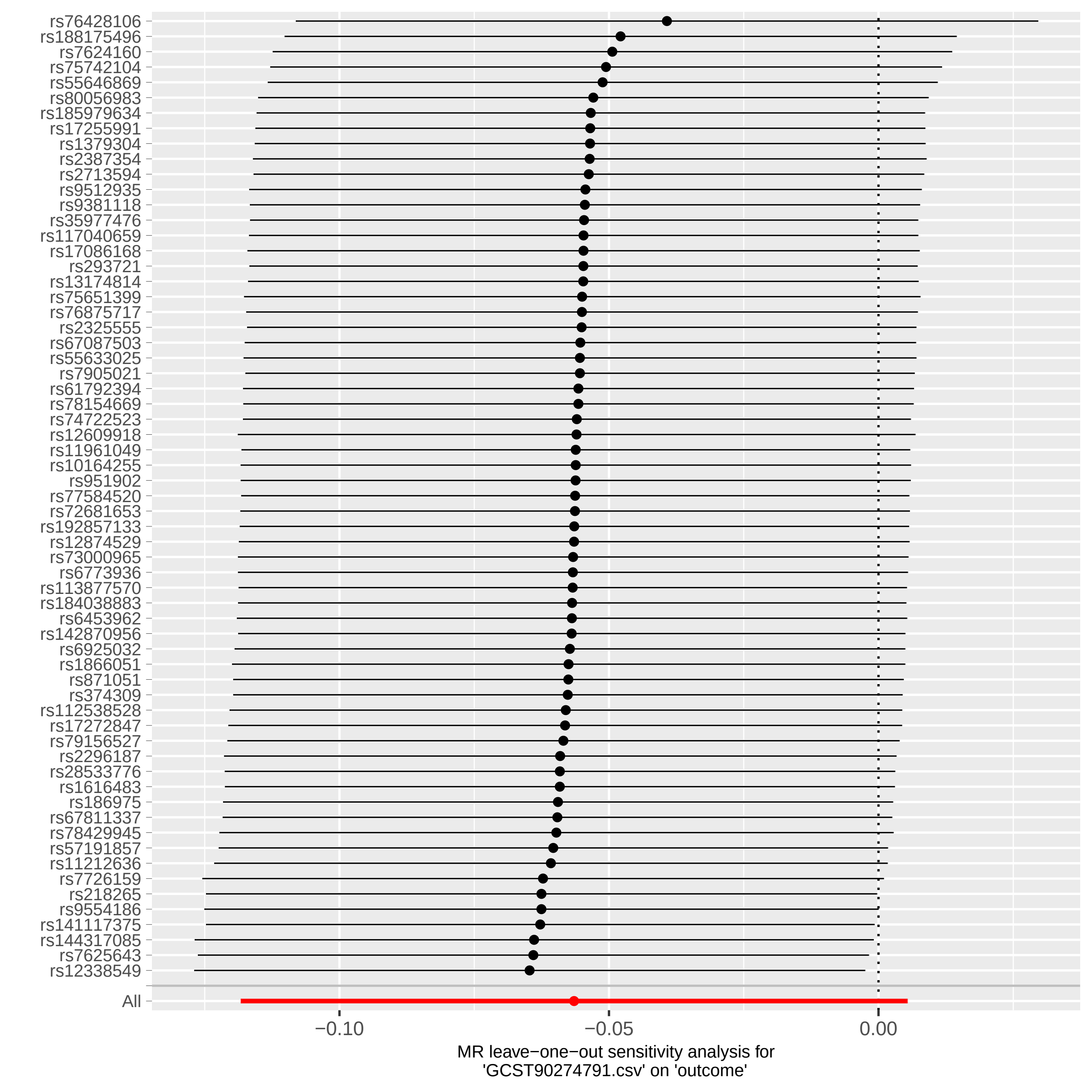

rs76428106
rs188175496
rs7624160
rs75742104
rs55646869
rs80056983
rs185979634
rs17255991
rs1379304
rs2387354
rs2713594
rs9512935
rs9381118
rs35977476
rs117040659
rs17086168
rs293721
rs13174814
rs75651399
rs76875717
rs2325555
rs67087503
rs55633025
rs7905021
rs61792394
rs78154669
rs74722523
rs12609918
rs11961049
rs10164255
rs951902
rs77584520
rs72681653
rs192857133
rs12874529
rs73000965
rs6773936
rs113877570
rs184038883
rs6453962
rs142870956
rs6925032
rs1866051
rs871051
rs374309
rs112538528
rs17272847
rs79156527
rs2296187
rs28533776
rs1616483
rs186975
rs67811337
rs78429945
rs57191857
rs11212636
rs7726159
rs218265
rs9554186
rs141117375
rs144317085
rs7625643
rs12338549
All
−0.10
−0.05
0.00
MR leave−one−out sensitivity analysis for
'GCST90274791.csv' on 'outcome'
